# Articular cartilage gene expression patterns in the tissue surrounding the impact site following applications of shear and axial loads

**DOI:** 10.1186/s12891-018-2374-2

**Published:** 2018-12-22

**Authors:** R. S. McCulloch, P. L. Mente, A. T. O’Nan, M. S. Ashwell

**Affiliations:** 1 Joint Department of Biomedical Engineering, North Carolina State University, Raleigh, NC, USA and University of North Carolina, Chapel Hill, North Carolina USA; 2 Department of Human Physiology, Gonzaga University, 502 E Boone Ave, Spokane, WA 99258 USA; 3 Department of Animal Science, North Carolina State University, 120 Broughton Dr, Raleigh, NC 27695 USA

**Keywords:** Osteoarthritis, Porcine, Articular injury

## Abstract

**Abstract:**

**Background:**

Osteoarthritis is a degradative joint disease found in humans and commercial swine which can develop from a number of factors, including prior joint trauma. An impact injury model was developed to deliver in vitro loads to disease-free porcine patellae in a model of OA.

**Methods:**

Axial impactions (2000 N normal) and shear impactions (500 N normal with induced shear forces) were delivered to 48 randomly assigned patellae. The patellae were then cultured for 0, 3, 7, or 14 days following the impact. Specimens in the tissue surrounding the loading site were harvested and expression of 18 OA related genes was studied via quantitative PCR. The selected genes were previously identified from published work and fell into four categories: cartilage matrix, degradative enzymes, inflammatory response, and apoptosis.

**Results:**

Type II collagen (*Col2a1*) showed significantly lower expression in shear vs. axial adjacent tissue at day 0 and 7 (fold changes of 0.40 & 0.19, respectively). In addition, higher expression of degradative enzymes and *Fas*, an apoptosis gene, was observed in the shear specimens.

**Conclusions:**

The results suggest that a more physiologically valid shear load may induce more damage to surrounding articular cartilage than a normal load alone.

**Electronic supplementary material:**

The online version of this article (10.1186/s12891-018-2374-2) contains supplementary material, which is available to authorized users.

## Background

Although osteoarthritis (OA) is the most common joint disorder in humans [1], the multi-factorial pathogenesis of OA is still not completely understood [2, 3]. Joint trauma, however, is a known causative factor in the development of OA [2, 4]. Controlled experimental injury models in an in vitro setting [5–11] provide the ability to precisely control loading to study the early stages of articular cartilage degradation in OA. Most impact studies use loading normal to the cartilage surface, however a realistic physiological trauma will most likely generate large shear forces. An axial load will cause a compression of the cartilage surface, and subsequent movement of fluid through the matrix following compression of the tissue. However, our work with three dimension load cells indicates that the while some shear loading is present at the surface, these forces are not large. A realistic trauma that may lead to OA, such as a fall, sports injury, car crash, would likely involve multi-axial loading with much higher shear forces. Thus, it was our aim to intentionally deliver higher shear loads to better model what may happen in a physiological trauma.

ACL transection models are used to study in vivo progression of OA [12], however these models induce general instability and make it hard to precisely control the degree of load changes. Other models include disuse models of OA [13], however these likely result in different pathways than that induced by a traumatic injury. Many models use cartilage explants that are removed from the surrounding tissue and then subjected to loading [14]. However, these models make it hard to differentiate whether the changes were due to the harvesting of the explant or the intentionally induced trauma.

Degenerative joint disease is not limited to only humans. Lameness is one of the primary reasons for culling in commercial swine, accounting for 22.5% of the culled sows in Southern China [15]. Structural lameness and leg weakness have been issues in commercial swine for decades and continues to contribute to sow longevity issues [16]. Kirk and coworkers found many of the locomotive problems found in sows were due to arthritis [17]. In our previous work (unpublished), we found that nearly 75% of the femoral heads from culled sows examined in a slaughterhouse have naturally occurring osteoarthritis at different stages of degeneration. Even in growing pigs, degenerative joint disease is a major cause of lameness [18], where trauma is a common cause of that lameness [19]. Because of the occurrence and similarities of OA in pigs, porcine models of OA have been used in both in vitro and in in vivo models to study OA and its treatments. [20–23]

In our previous work, we developed a shear injury model of OA and evaluated gene expression changes in the articular cartilage directly below the impact injury site [24]. In the present work, expression changes in the tissue adjacent to the loading site were evaluated. It was hypothesized that shear impacts would generate more degradative changes to adjacent tissue than normal impacts.

## Methods

**Fig. 1:**
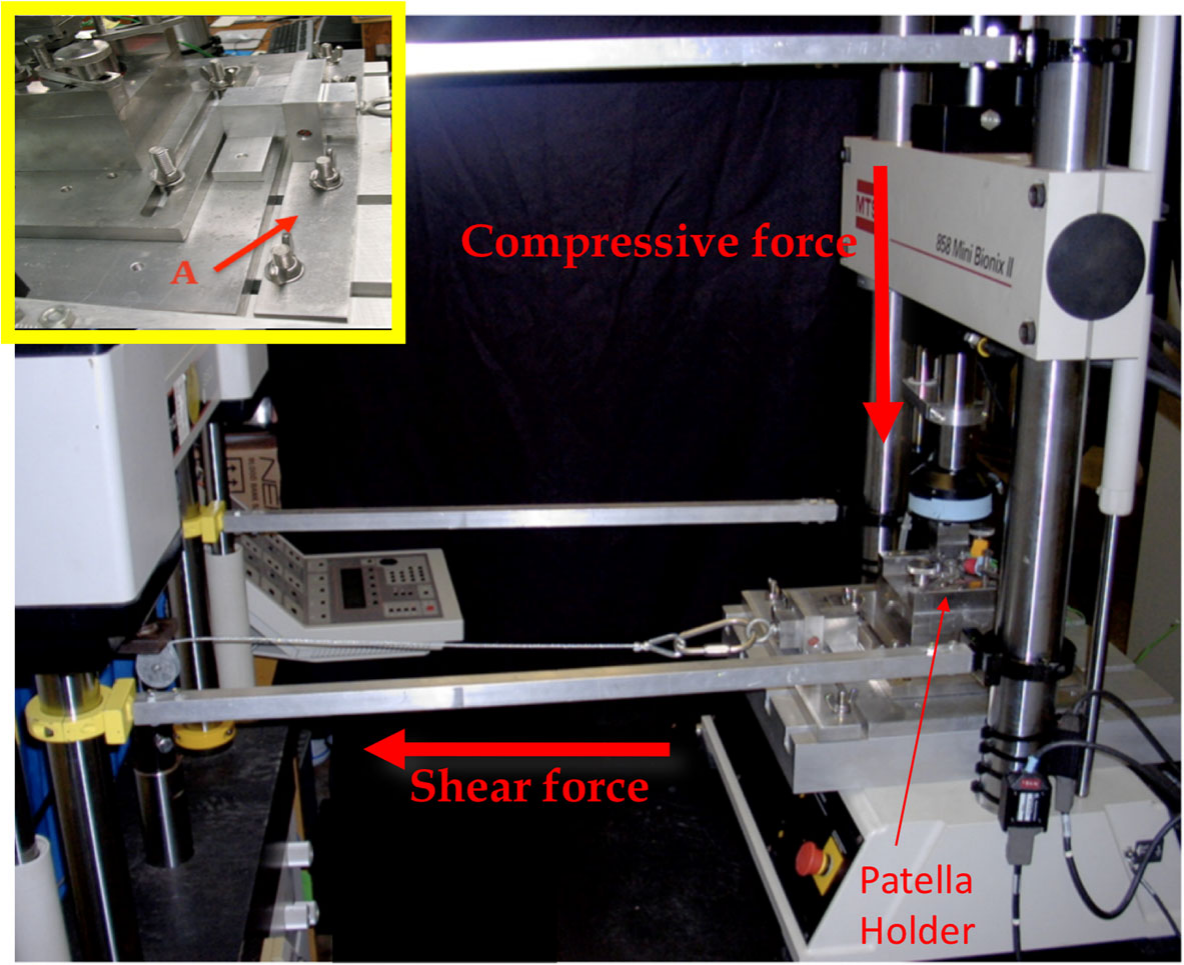
Mechanical impact testing setup. The patella holder can be seen to the right, positioned in the first load frame. For pure axial impactions, the cross-head of the servo-hydraulic load frame on the right delivered an orthogonal load to the patella facet. For impactions with elevated shear, the load frame on the right delivered an axial impaction, while the load frame to the left displaced the patella tangentially 10 mm to induce elevated shear forces. The stop bar in the inset image shows the stop bar (A) that limited tangential movement to 10 mm

Forty-eight (48) intact porcine knee joints were obtained from a local slaughterhouse and the patellae were sterilely removed. In a custom testing apparatus using a servo-hydraulic load frame, an impact was delivered to each patella orthogonal to the articular surface via a stainless-steel impactor of 10 mm radius and 10 mm length. A custom holder was manufactured for the patellae, to position them with the facet orthogonal to the direction of the axial impact. The holder had a spherical depression, and the patellae were potted in sterile Polymethylmethacrylate (PMMA - bone cement) in a spherical shape to allow for proper positioning and orientation of the patellae. The axial impaction (axial) delivered a load of 2000 N normal to the surface [7]. For the shear impaction (shear), once a 500 N normal load was reached, the patella was immediately mechanically displaced (via a second load frame) using a cable and pulley arrangement 10 mm tangentially to induce shear forces in the cartilage, resulting in mean shear forces at the impactor tip of 198 ± 59.2 N (Fig. 1). Additionally, a set of 24 control specimens were processed in the same manner, but without impacts.

**Fig. 2:**
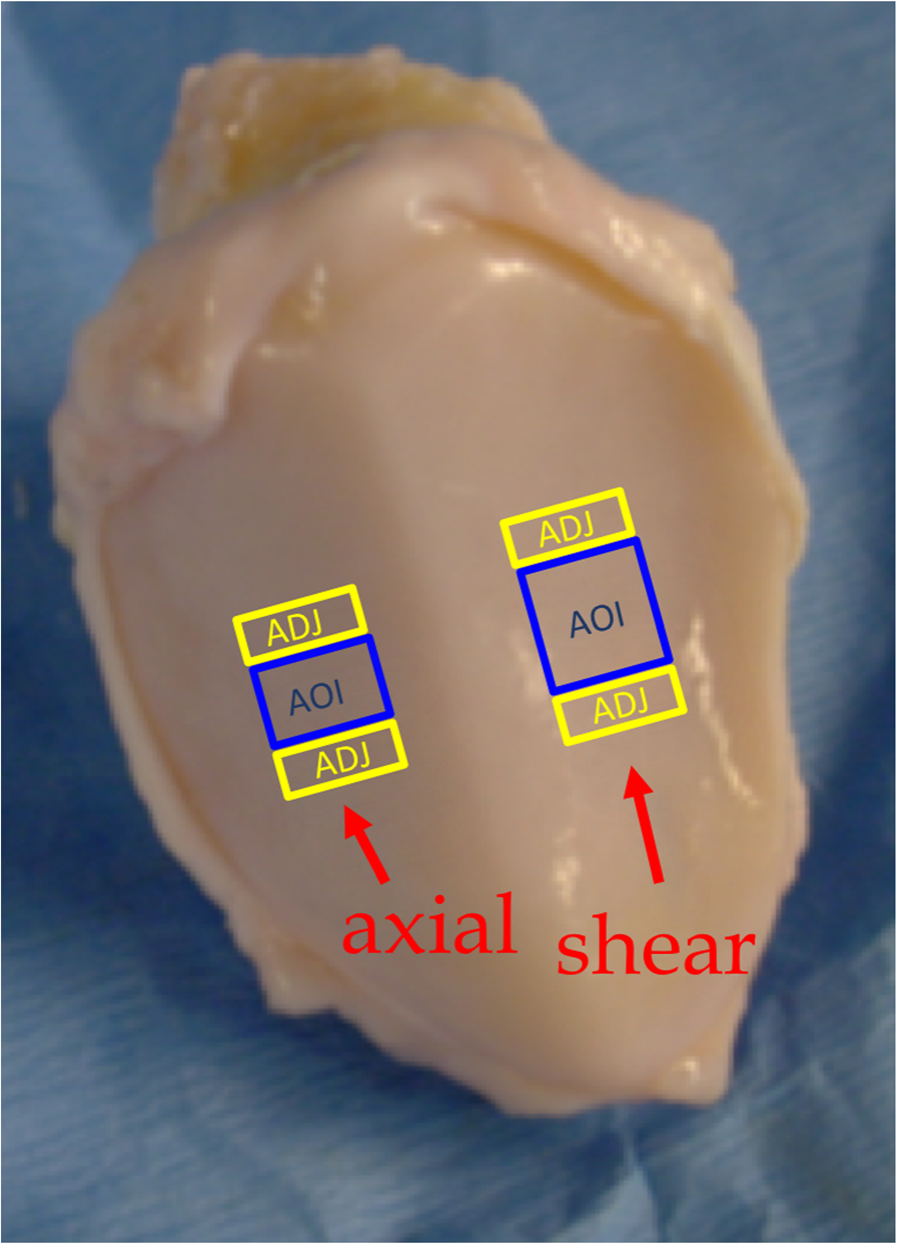
Specimen locations. Full-thickness cartilage specimens were harvested from the location directly below the impact site (AOI sample in blue), and in the tissue adjacent to the impaction site (ADJ sample in yellow). The relative size of the axial specimens is shown on the left, and the shear on the right. This figure is for demonstration only, actual patellae were given identical treatments on each facet

After impact, the patellae were placed into culture (Culture media: Dulbecco’s MEM/Ham’s F12 with 10% fetal calf serum, ascorbic acid (25 μg/ml) with penicillin 100 units/ml, streptomycin 100 μg/ml, and amphotericin B 25 μg/ml) for 0, 3, 7, or 14 days. At each time point, full thickness slices of cartilage were harvested at the area of impact (AOI) and from tissue adjacent to the impact site (ADJ). The samples were flash frozen in liquid N_2_ and stored at − 80 °C. The AOI specimens for the axial impactions were 5 × 10 mm, to represent the area under the impactor tip contact, and the AOI specimens for the shear impactions were 10 × 10 mm to accommodate the tangential displacement of the impactor during loading. In each case, ADJ specimens of 3 × 10 mm were harvested from the proximal and distal ends of the impaction sites (Fig. 2).

Gene expression analysis was completed using methods previously described [24, 25], briefly: RNA was extracted using Tri Reagent (Molecular Research Center Inc., Cincinnati, OH) after grinding the cartilage specimens to a fine powder. RNA purity was assessed (Nanodrop-1000, Thermo Scientific, Wilmington, DE), and was then reverse transcribed (High Capacity cDNA Reverse Transcription Kit, Applied Biosystems Inc., Foster City, CA). Quantitative real-time PCR (qPCR) was performed, and the genes of interest were normalized to a panel of 4 previously identified housekeeping genes [25]. See Table 1 regarding full gene and primer information, and please refer to Additional file 1 for primer design and qPCR analysis. Eighteen genes associated with early stage OA were evaluated: 1) Cartilage Matrix: *Col1a1, Col2a1, Acan, Sox9, Opn, Comp;* 2) Degradative Enzymes and Inhibitors: *Mmp1, Mmp3, Mmp13, Timp1, Timp2, Adamts5;*3) Inflammatory Response and Signaling: *Ihh, Tgfb, Inos, Chi3l1;* and 4) *Apoptosis*: *Casp8, Fas.*

**Table 1:** Gene primer information

Gene Name	Sequence (5′ - > 3′)	Annealing Temp	Amplicon length	NCBI Number
*Cartilage Matrix*				
Collagen, Type I, Alpha 1 (*Col1a1*)	F: CAACCGCTTCACCTACAGC			
	R: TTTTGTATTCGATCACTGTCTTGCC	60	101	AK236626
Collagen, Type II, Alpha 1 (*Col2a1)*	F: GAGAGGTCTTCCTGGCAAAG			
	R: AAGTCCCTGGAAGCCAGAT	60	118	AF201724.1
Aggrecan (*Acan)*	F: TGCAGGTGACCATGGCC			
	R: CGGTAATGGAACACAACCCCT	60	79	AF201722b
SRY (sex determining gene region Y) box-9 (*Sox9)*	F: CAGGGCTCTGTGCTCTACTCC			
	R: GGGTTACGGTCTTTCTTCGGT	60	230	NM_213843.1
Osteopontin (*Opn)*	F: CCGCAGCCAGGAGCAGTC			
	R: GTTGATCTCAGAAGACGCACTCTC	55	214	NM_214023.1
Cartilage oligometric matrix protein (*Comp)*	F: GGCTGGAAGGACAAGACATC			
	R: CCTCATAGAACCGCACTCTG	55	82	XM_003123529.1
*Degradative Enzymes & Inhibitors*				
Matrix metalloprotease-1 (*Mmp1)*	F: TGATGGACCTGGAGGAAACC			
	R: GAGCAGCCACACGATACAAG	59	131	NM_001166229
Matrix metalloprotease-3 (*Mmp3)*	F: GATGTTGGTTACTTCAGCAC			
	R: ATCATTATGTCAGCCTCTCC	50	197	NM_001166308.1
Matrix metalloprotease-13 (*Mmp13)*	F: CCAAAGGCTACAACTTGTTTCTTG			
	R: TGGGTCCTTGGAGTGGTCAA	60	77	AF069643
TIMP Metallopeptidase Inhibitor-1 (*Timp1)*	F: CCTCGTACCAGCGTTATG			
	R: CGTTCCACAGTTGTCCAG	59	177	NM_213857.1
TIMP Metallopeptidase Inhibitor-2 (*Timp2)*	F: ATATACGAGAACACCAGACC			
	R: GGAATGATTACAACGGATGC	59	152	AK237154.1
ADAM Metallopeptidase with Thrombospondin Type 1 Motif 5 (*Adamts5)*	F: CGCTGCCACCACACTCAA			
	R: CGTAGTGCTCCTCATGGTCATCT	60	80	NM_007038.3
*Inflammatory Response*				
Indian Hedgehog (*Ihh)*	F: CAGCGGGCGCTATGAAGGCA			
	R: GGTCCTTGCAGCGCTGGGTC	60	140	XM_001925486.1
Transforming growth factor β (*Tgfb)*	F: GGAGTGGCTGTCCTTTGATGT			
	R: AGTGTGTTATCTTTGCTGTCA	60	117	NM_214015.1
nitric oxide synthase 2, inducible (*Inos)*	F: TGAATTTGTCAACCTGTATTAC			
	R: CTTTGTTACCGCTTCCAC	53	82	NM_001143690.1
Chitinase-3-like protein 1 (*Chi3l1)*	F: TGACGCTCTATGACACAC			
	R: GGCTAGGTCCAGTCCATC	62	194	NM_001001540
*Cell Proliferation and Apoptosis*				
Caspase-8 (*Casp8)*	F: TGGGCAAACAGATGCCACAACCT			
	R: CCCCTTCAATCTAGCCCACCCCC	60	153	NM_001031779.2
Fas (TNF receptor superfamily, member 6) (*Fas)*	F: TAGAGTTTGTGATGGAGAA			
	R: ATTGAGAAGTGTGACAGA	53	107	NM_213839.1

Full names with abbreviations, primer sequences, annealing temperatures, amplicon length, and NCBI numbers

The relative gene expression levels were compared between the ADJ specimens for axial and shear impacts, and between ADJ and their associated AOI specimens. Differences in AOI specimens were reported previously [24]. A linear mixed model (SAS, SAS Institute, Cary, NC) was used to evaluate differences in fold changes, following the methods of Steibel et al. [26]. The family-wise error rate was controlled using the false discovery rate method (FDR) [27] to generate a *q-*value. Because each time point/impact combination had a small number of samples, the q-value significance threshold was set at *q* < 0.2. This allowed detection of changes without being overly restrictive, and due to how FDR controls for error in results deemed significant, a threshold of up to 0.5 may be acceptable [27].

## Results

**Fig. 3:**
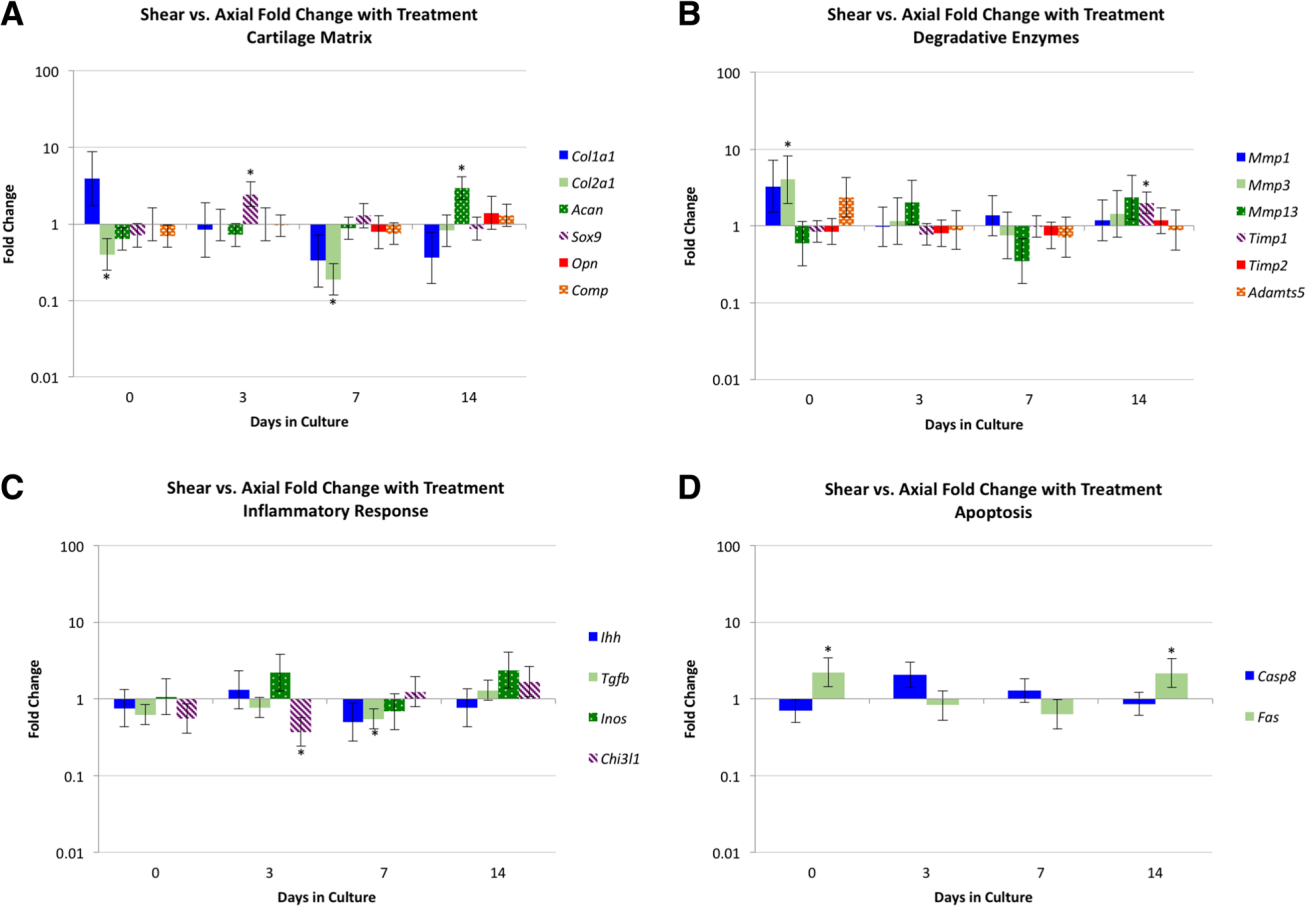
Fold changes for shear vs. axial adjacent (ADJ) tissue. The vertical axis indicates fold change as a log scale, and the horizontal axis indicates days in culture. The error bars depict standard error, and significant differences are indicated with an asterisk (*). Panels: **a**) cartilage matrix, **b**) degradative enzymes, **c**) inflammatory markers, and **d**) apoptosis

The 72 patellae (36 right, 36 left) were randomly assigned to a treatment and time point, with 6 patellae at each combination (axial, shear, control; and 0, 3, 7, 14 days in culture). Fold changes (FC) were compared between shear and axial ADJ tissue (Fig. 3). In the cartilage matrix, *Col2a1* expression was significantly lower in shear vs. axial specimens at day 0 (FC 0.40) and day 7 (FC 0.19). *Sox9* was higher in shear specimens on day 3 (FC 2.46), and *Acan* was higher in shear specimens on day 14 (FC 2.96). In the degradative enzymes, *Mmp3* was elevated on day 0 (FC 4.05), whereas *Timp1* was elevated on day 14 (FC 2.01) in the shear samples. For the inflammatory response genes, expression of *Chi3l1* was lower in shear specimens on day 3 (FC 0.37), and *Tgfb* was lower in shear specimens on day 7 (FC 0.55). For the apoptosis related genes, *Fas* demonstrated higher expression in the shear specimens at day 0 (FC 2.22) and day 14 (FC 2.19).

The ADJ tissue specimens were also compared to their associated AOI specimens to examine fold change differences at each time point (Table 2). *Col1a1* was elevated in both axial and shear ADJ tissue at day 0. However, *Col2a1* showed lower expression in shear ADJ tissue on day 7. *Mmp3* and *Adamts5* were both more highly expressed in the shear ADJ specimens at the last time point. *Casp8* was lower in shear ADJ vs AOI tissue on both day 0 and day 14.

**Table 2:** Differential gene expression between the tissue adjacent to the impact site (ADJ) and the area of impact (AOI) at each time point for each type of impact (axial and shear)

Gene	Fold Changes				Fold Changes			
	Axial ADJ vs. AOI				Shear ADJ vs. AOI			
	Day 0	Day 3	Day 7	Day 14	Day 0	Day 3	Day 7	Day 14
Cartilage Matrix								
*Col1a1*	**13.50**	2.36	1.53	**0.22**	**10.03**	0.40	0.93	0.54
*Col2a1*	0.78	1.42	0.81	1.03	0.57	0.57	**0.37**	0.78
*Acan*	1.06	1.26	0.96	0.73	1.03	0.78	0.70	1.04
*Sox9*	0.62	**0.49**	0.82	1.31	**0.50**	0.96	1.04	**0.39**
*Opn*	**2.89**	1.19	1.06	**2.75**	**2.99**	0.87	0.66	1.46
*Comp*	0.87	0.69	0.90	1.07	1.48	0.71	0.90	0.97
Degradative Enzymes & Inhibitors								
*Mmp1*	0.55	1.26	0.49	1.05	0.64	1.10	1.37	3.38
*Mmp3*	0.52	2.23	0.70	1.90	0.89	1.60	**0.16**	**4.90**
*Mmp13*	0.96	1.12	0.88	0.81	1.67	0.42	1.20	2.70
*Timp1*	1.45	0.85	0.77	0.87	0.92	0.74	0.95	1.41
*Timp2*	**2.44**	**2.73**	0.91	1.76	0.78	0.94	0.97	1.31
*Adamts5*	1.84	1.81	0.94	0.86	2.17	1.07	0.81	**5.24**
Inflammatory Response & Signaling								
*Ihh*	1.04	**3.53**	1.85	0.74	**0.39**	1.69	**0.41**	**0.34**
*Tgfb*	1.27	0.98	1.24	1.46	1.54	1.20	0.76	1.10
*Inos*	0.36	0.87	1.39	1.18	1.11	1.15	1.54	1.35
*Chi3l1*	1.00	0.93	0.96	0.75	0.51	0.63	1.14	0.79
Cell Proliferation & Apoptosis								
*Casp8*	0.63	0.56	0.84	1.34	**0.50**	1.01	1.05	**0.39**
*Fas*	**0.37**	1.36	1.70	1.24	1.95	1.24	0.58	1.49

Significant *q*-values (*q* < .2) are indicated by bold type

**Fig. 4:**
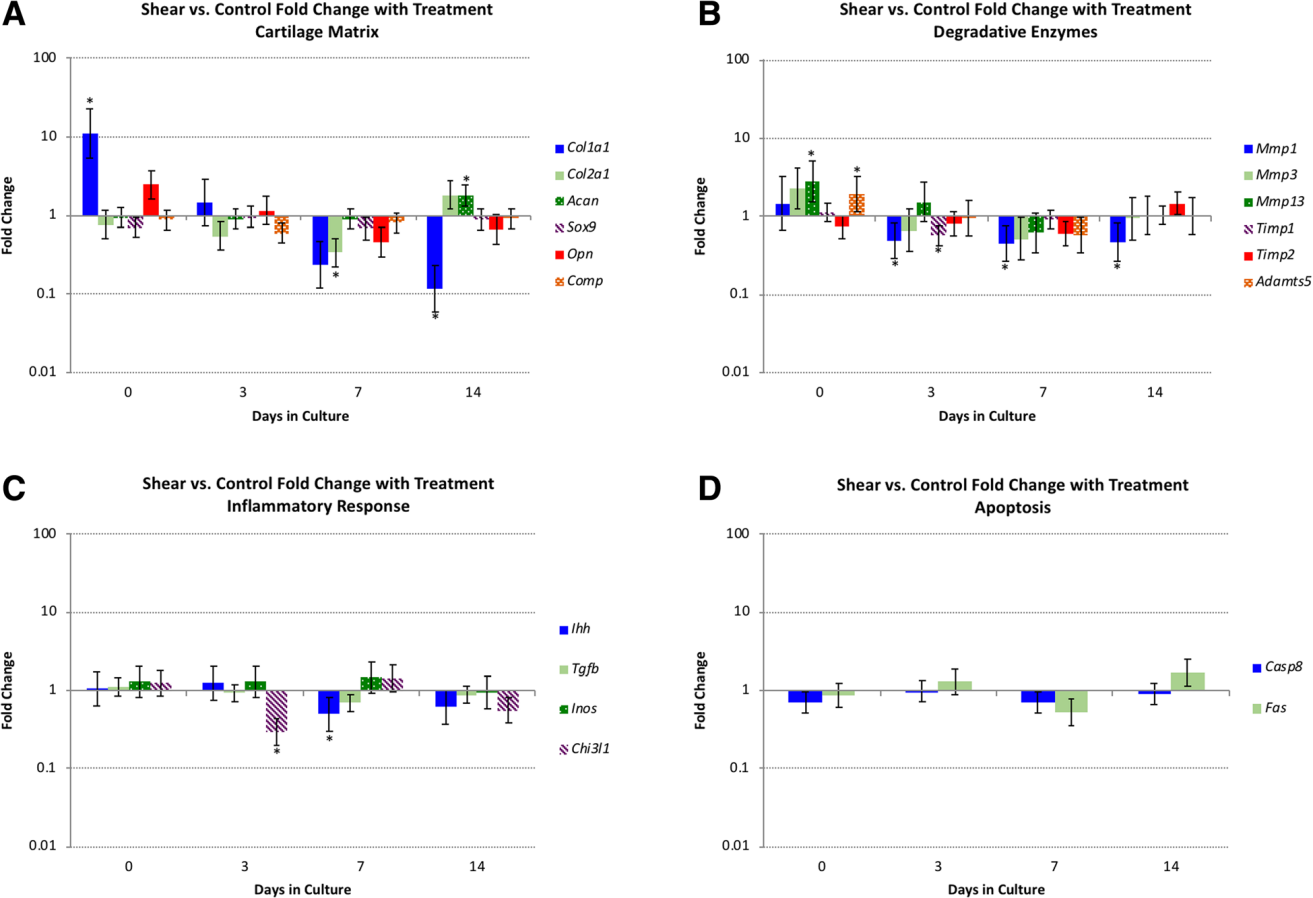
Fold changes for shear adjacent (ADJ) vs. control tissue. The vertical axis indicates fold change as a log scale, and the horizontal axis indicates days in culture. The error bars depict standard error, and significant differences are indicated with an asterisk (*). Panels: **a**) cartilage matrix, **b**) degradative enzymes, **c**) inflammatory markers, and **d**) apoptosis

Finally, shear ADJ specimens were compared to control specimens at their associated time points (Fig. 4). *Col1a1* was more highly expressed in shear specimens on day 0 vs control (FC 11.22) while it demonstrated lower expression on day 14 (FC 0.12). On day 7 *Col2a1* showed lower expression in the shear specimens (FC 0.34). *Mmp1* showed lower expression than control on day 3,7, & 14 (FC 0.49, 0.45, & 0.47 respectively). *Mmp-13* showed higher expression on day 0 in the shear specimens (FC 2.82). *Timp-1* was demonstrated lower expressed in shear specimens on day 3 (FC .57). *Adamts-5* was more highly expressed in shear specimens on day 0 (FC 1.97). *Ihh* showed lower expression in shear specimens on day 7 (FC 0.50). *Chi 3 l1* was less expressed in shear specimens compared to control on day 3 (FC 0.30).

## Discussion

The goal of this study was to compare two in vitro loading scenarios, one with primarily normal loading, and another with elevated shear loading. The elevated shear loading is likely more representative of true physiological loading. It was believed that shear loading would induce more deleterious effects in the adjacent non-impacted tissue. Our results suggest that this was the case, with *Col2a1* downregulated in shear ADJ tissue, *Mmps* showing higher expression, and elevated levels of *Fas* expression.

Though *Col1a1* levels were elevated early in shear vs axial, the difference was not significant, and the expression did not remain elevated. However, *Col2a1* remained lower at all time points in shear ADJ tissue, and tended to be lower than shear AOI. Similarly, Col1a1 was more highly expressed in shear specimens vs. control, and Col2a1 showed lower expression in the shear specimens vs. control. This finding is notable, as *Col2a1* is the primary collagen found in articular cartilage, and its depression may indicate that the chondrocytes are not effectively repairing the damage, especially when taken in light of the more highly expressed levels of *Col1a1*. This could suggest that the chondrocytes are reverting to a dedifferentiated state following the loading [8, 28, 29]. The elevation of *Sox9* and *Acan* indicates a repair effort is being mounted [30]. Taken together with the collagen expression differences, the cells may be attempting recovery from the trauma, but with responses that are ineffective for proper tissue repair.

The chondrocytes show elevated levels of degradative enzymes, and at later time points, elevated levels of an associated inhibitor, consistent with the belief that a repair attempt is being mounted. Early elevation of degradative enzymes (*Mmps*) in shear vs axial ADJ and in shear ADJ vs AOI, and in shear ADJ vs. control indicates a chondrocyte response to the trauma and attempt to effect repairs, and suggests that shear loads are more damaging, likely due to additional forceful fluid movement through the matrix. This is consistent with other work that has found elevated levels of *Mmps* in early OA [10, 31]. Also, *Adamts5*, an aggrecanase, was elevated more in the shear ADJ vs AOI tissue at the last time point, which suggests more matrix breakdown in the shear adjacent tissue. Following the early elevated degradative enzyme expression, the increase of *timp1* (an inhibitor of *Mmps)* at the last time point may suggest the chondrocytes are attempting to limit matrix breakdown, consistent with the idea that the cells are attempting repairs via initial breakdown that is stemmed at later points following the injury.

*Ihh* has been associated with chondrocyte hypertrophy and has been found to be elevated in OA [32], and its lower expression in our shear ADJ vs AOI tissue may be the result of the aforementioned dedifferentiation of the chondrocytes, therefore generating inconsistent and ineffective repair efforts. *Fas* is associated with apoptosis, and its elevation in shear vs axial ADJ tissue may indicate more cell death, in-line with work showing its increase at OA lesions [33].

## Conclusions

If we can gain a better understanding of the very early degenerative changes that precede full blown OA we will be better able to identify potential targets for therapeutic intervention, treat the disease, and prevent the debilitating changes that occur further down the road, having dual benefit in human medicine as well as sustainable pork production. The results presented here show the effects on surrounding tissue when a more realistic loading model is used to simulate OA in vitro. Our findings suggest that shear loads may be more damaging to surrounding tissue than a normal load alone. In the shear specimens, the adjacent tissue showed reduced levels of appropriate collagen expression, and increased expression of degradative enzymes and an apoptosis related gene. This suggests chondrocytes in the shear tissue responded with attempts at repairs but with an ineffective response.

## Additional file

Additional file 1: Contains details of primer design and the qPCR procedure used for this study. (DOCX 32 kb)
