# Supplementary material for: Articular cartilage gene expression patterns in the tissue surrounding the impact site following applications of shear and axial loads
Source: BMC Musculoskelet Disord. 2018 Dec 22;19:449. doi: 10.1186/s12891-018-2374-2 (PMC6303924; doi:10.1186/s12891-018-2374-2)
Supplement: Supplementary file 1 — Contains details of primer design and the qPCR procedure used for this study. (DOCX 32 kb) [file 12891_2018_2374_MOESM1_ESM.docx]

**Additional File 1**

**Title**

Articular cartilage gene expression patterns in the tissue surrounding the impact site following applications of shear and axial loads

**Authors**

R. S. McCulloch, Ph.D.; P. L. Mente, Ph.D.; A.T. O’Nan, M.S.; M.S. Ashwell, Ph.D.

**Genes**

Full gene names, with abbreviations are provided in Table S1.

**Primer Design**

Primer pairs were designed using Beacon Designer (Premier Biosoft International, Palo Alto, CA) to be used with SYBR Green I Master Mix. Primers were designed from porcine gene sequences where possible. When not available, primers were designed using conserved regions of human, bovine, or canine sequences. They were designed to cross an intron-exon boundary (Table S1).

**qPCR Procedure**

The qPCR procedure:

- Performed in 20 μL reaction:
  - 1 μL of diluted cDNA
  - 400 nM of forward and reverse primers
  - 10 nM fluorescein (as a reference dye)
  - 0.5 μL of 1X Power SYBR Green I Master Mix.
- 3-step amplification protocol, performed in an iCycler IQ (Bio-Rad, Hercules, CA):
  - Denaturation with one cycle at 95°C for 7 minutes
  - 40 cycles of 30 sec at 95°C for denaturation
  - 30 sec at 50°-62°C for annealing
  - Extension for 30 sec at 72°C
  - Product melting cycle of 5 min at 72°C, 1 min at 95°C
  - 1 min at 55°C.
- Samples were amplified in triplicate
- Reaction efficiency for each primer set assessed using standard curves via a dilution series (iCycler iQ Real-Time PCR Detection System Software)
- Gene target specificity of the reactions evaluated with a melt curve generated at the end of the PCR amplification cycle
- One cDNA product from each primer pair was sequenced to verify that the PCR product corresponded to the intended gene
- Expression for the OA related genes was normalized to the geometric mean of 4 genes identified in our previous work as being the most stable in tissues given our treatment protocol [1]
- Housekeeping genes: *Actb, Gapdh, Sdha, and Ppia*.

[1] McCulloch RS, Ashwell MS, T O’Nan A, Mente PL (2012) Identification of stable normalization genes for quantitative real-time PCR in porcine articular cartilage. J Anim Sci Biotechnol 3:36.
